# Characterization of *Alternaria* and *Colletotrichum* Species Associated with Pomegranate (*Punica* *granatum* L.) in Maharashtra State of India

**DOI:** 10.3390/jof8101040

**Published:** 2022-09-30

**Authors:** Nanjundappa Manjunatha, Jyotsana Sharma, Somnath S. Pokhare, Ruchi Agarrwal, Prakash G. Patil, Jaydip D. Sirsat, Mansi G. Chakranarayan, Aarti Bicchal, Anmol S. Ukale, Rajiv A. Marathe

**Affiliations:** ICAR-National Research Centre on Pomegranate, Solapur 413255, Maharashtra, India

**Keywords:** *Alternaria alternata*, *Alternaria burnsii*, heart rot, *Colletotrichum gloeosporioides* species complex, *Colletotrichum* species, anthracnose

## Abstract

Fungal pathogens are a major constraint affecting the quality of pomegranate production around the world. Among them, *Alternaria* and *Colletotrichum* species cause leaf spot, fruit spot or heart rot (black rot), and fruit rot (anthracnose) or calyx end rot, respectively. Accurate identification of disease-causing fungal species is essential for developing suitable management practices. Therefore, characterization of *Alternaria* and *Colletotrichum* isolates representing different geographical regions, predominantly Maharashtra—the Indian hub of pomegranate production and export—was carried out. Fungal isolates could not be identified based on morphological characteristics alone, hence were subjected to multi-gene phylogeny for their accurate identification. Based on a maximum likelihood phylogenetic tree, *Alternaria* isolates were identified as within the *A. alternata* species complex and as *A. burnsii*, while *Colletotrichum* isolates showed genetic closeness to various species within the *C. gloeosporioides* species complex. Thus, the current study reports for the first time that, in India, the fruit rots of pomegranate are caused by multiple species and not a single species of *Alternaria* and *Colletotrichum* alone. Since different species have different epidemiology and sensitivity toward the commercially available and routinely applied fungicides, the precise knowledge of the diverse species infecting pomegranate, as provided by the current study, is the first step towards devising better management strategies.

## 1. Introduction

Pomegranate (*Punica granatum* L.) has been cultivated as a fruit crop since ancient times. It produces edible fruits with innumerable health benefits and high commercial value. Moreover, in recent years, possible applications of extracts of pomegranate fruit peel as natural pesticides or food preservatives have also been envisaged [1,2,3]. Over the last few decades, global market demand of pomegranate fruit has increased remarkably, resulting in alluring monetary returns to growers and a constant increase in cultivation area and production of this horticultural crop, especially in India. Globally, India is the largest pomegranate producer, with more than 41% of the world’s area and production. Currently, in India, the crop occupies an area of 288,000 ha, with a production of 3,256,000 tons [4]. Maharashtra state is the first pomegranate producer in India with a cultivated area of 171,000 ha and a production of 1,795,000 MT, as well as the first exporter, with 51,699 MT and a value of INR 3520 million in 2020–2021 [5], with a share of 59.4% in area, 55.13% in production, and 84.41% in export at the national level. In Maharashtra, the largest area cultivated with pomegranate (47,380 ha) is in the Solapur district. Due to the unique properties of pomegranate produced in Solapur, it has been awarded the geographical indication tag and more than 80% of planting material is now supplied from Maharashtra to other pomegranate growing states of the country.

In India, among all the commercial pomegranate cultivars, cv. Bhagawa is the most popular; it accounts for more than 86% of the pomegranate production area and is requested in local and export markets. However, this cultivar is highly susceptible to several fungal pathogens, among which *Alternaria* and *Colletotrichum* species are the major pathogens affecting the quality of pomegranate production [6,7]. Under changing climatic conditions, in recent times, infections by these pathogens are becoming more frequent in India [8,9]. *Alternaria* species cause leaf spot, fruit spot and heart rot (black rot) of pomegranate, whereas *Colletotrichum* species cause a fruit rot called anthracnose/calyx end rot [7,8,9]. These fungal pathogens cause huge economic losses to growers, as infected fruits become unmarketable. However, the precise estimation of losses caused by heart rot and anthracnose to pomegranate production in India is not available, though in 2020–2021, farmers in Maharashtra reported up to 50% losses due to these pathogens.

In the absence of resistant cultivars, fungicide application is the most widespread management method of fungal plant diseases. However, due to overuse, fungicide resistance may develop in the fungal pathogen populations. Fungicide resistance of *Alternaria* and *Colletotrichum* sp. to different fungicides was reported [10,11] earlier. Moreover, within the same genus of plant pathogenic fungi, isolates may exhibit differences in their sensitivity to various fungicides. For example, some species of *Colletotrichum* are inherently resistant to benomyl and, thus, their baseline sensitivity has been used to separate species complexes [12,13,14,15]. The accurate species differentiation is crucial for the implementation of suitable management practices and may have quarantine significance [16,17].

Identification of plant pathogenic fungi at the species level has traditionally relied mainly on morphological and cultural characteristics. However, these traditional techniques are limited in their effectiveness as growth medium and temperature are known to cause variation in cultural and morphological characteristics [18]. Therefore, in the last few years, DNA sequence-based identification has also been widely used. Sequences of the internal transcribed spacer (*ITS*; *ITS1/ITS4* region 5.8S) [19] are the most commonly used barcode loci for fungi; however, they alone are not always able to discriminate species of plant pathogenic fungi [20]. Multi-locus phylogenetic analyses have proven to be more reliable in addressing the challenge of identifying pathogenic fungal species [18,20]. In addition to ITS, protein-coding loci, such as Translation Elongation factor-1 (*TEF-1*), glyceraldehyde-3-phosphate dehydrogenase (*GAPDH*) and actin (*ACT*), have been commonly used to resolve pathogenic fungal species infecting pomegranate, apple and citrus and other host plants [21,22,23].

Different species of *Alternaria* (*A. alternata, A. arborescens, A. gaisen, A. mali* and *A. tenuissima*) and *Colletotrichum* (*C. gloeosporioides, C. fioriniae, C. nymphaeae, C. siamense*, *C. simmondsii* and *C. theobromicola*) associated with heart rot and anthracnose/calyx end rot, respectively, have been reported from different pomegranate-growing regions worldwide [4,24,25,26,27,28]. *Alternaria* and *Colletotrichum* species are also known to cause diseases in several fruit and vegetable crops worldwide [29,30]. Consequently, cross infections of *Alternaria* and *Colletotrichum* species have been reported in many fruit crops [31,32,33]. Although it is a common practice of cultivating other horticultural crops in close proximity to pomegranate, it is, however, unknown if cross infections by *Alternaria* and *Colletotrichum* species occur between pomegranate and these crops.

In India, currently, Alternaria leaf spot, fruit spot and heart rot of pomegranate are imputed to *A. alternata* [7,9,30], and fruit anthracnose to *C. gleosporoides* [7,8,34,35,36]. Moreover, there are no studies available on species diversity and/or molecular characterization of *Alternaria* and *Colletotrichum* associated with pomegranate in India. Based on the findings of recent studies in other pomegranate-growing countries, we hypothesized that different species of these two fungal genera may be involved in the etiology of pomegranate fruit rots. Thus, the main objective of the present study was to identify and characterize *Alternaria* and *Colletotrichum* species associated with pomegranate fruit in India.

## 2. Materials and Methods

### 2.1. Sample Collection and Fungus Isolation

*Alternaria* and *Colletotrichum* isolates characterized in this study were isolated from symptomatic fruits and leaves of pomegranate collected from several orchards in different geographical regions of India, such as Maharashtra (MH), Karnataka (KA), Uttar Pradesh (UP), Madhya Pradesh (MP) and Tamil Nadu (TN), during 2015–2021 (Supplementary Table S1). For sample collection, places that were at least 100–150 km apart were included. Symptomatic tissues were excised, disinfected with 1% sodium hypochlorite (NaOCl) and thereafter rinsed three times with sterile distilled water. The sterile tissues were then placed on sterile potato dextrose agar (PDA, HiMedia Laboratories Pvt Ltd., Mumbai, India) medium with pH 7 and incubated at 24 ± 1 °C. All the isolates were purified by the hyphal tip technique and stored at 4 °C in mineral oil until further use. Of all the isolates, 12 *Alternaria* and 19 *Colletotrichum* isolates representative of the entire variability, were used for further characterization (Supplementary Table S1).

### 2.2. Morphological Characterization

Representative fungal isolates of *Alternaria* and *Colletotrichum* were grown on PDA amended with streptomycin sulphate (100 mg/L) at 25 ± 1 °C. Seven days after incubation, all the isolates were subjected to macroscopic and microscopic study by using a compound microscope (Nikon Eclipse 90*i*). Growth rate, colony appearance and conidium characteristics were recorded for each isolate of *Alternaria* and *Colletotrichum* according to the method previously described [21,37]. Statistical analyses of the data obtained were performed using WASP (Web Agri Stats Package) software available at https://ccari.icar.gov.in/waspnew.html (accessed on 30 June 2022). Multivariate statistical analysis, such as Principal Component Analysis, was performed using morphological data such as length and width of conidia.

### 2.3. Molecular Characterization

A multi-locus approach was employed to characterize the selected isolates of *Alternaria* and *Colletotrichum*. The barcoding genetic regions, such as ITS, LSU, NS and *TEF-1* for *Alternaria* and ITS*, GADPH* and *ACT* for *Colletotrichum*, were PCR amplified and sequenced for characterization (Table 1). For genomic DNA isolation, mycelium was harvested from colonies of fungal isolates grown on PDA after 7 days of incubation at 24 ± 1 °C. Genomic DNA was extracted using the CTAB method described earlier [38] with some modifications. PCR was carried out in a 10 μL reaction mixture containing 1 μL of 50 ng g DNA, 0.25 μL of 10 µM primer forward and reverse each (Table 1), 4 µL of 2X PCR master mix (HiMedia Laboratories Pvt Ltd., Mumbai, India) and 4.5 μL molecular-grade sterile water. PCR was performed in a Thermocycler (HiMedia, Laboratories Pvt Ltd., Mumbai, India) with the following PCR program for ITS, LSU, NS and *TEF-α* amplification: initial denaturation at 95 °C for 3 min, followed by 35 cycles of denaturation (95 °C for 30 s), annealing (55 °C for 30 s), extension (72 °C for 30 s) and the final extension at 72 °C for 7 min. Amplification of *GAPDH* and *ACT* was performed using touchdown PCR with an initial denaturation at 94 °C for 5 min, followed by 16 cycles of denaturation (94 °C for 30 s), annealing (60 °C for 30 s) and extension (72 °C for 45 s), followed by 25 cycles of denaturation (94 °C for 30 s), annealing (55 °C for 30 s), extension (72 °C for 45 s) and the final extension at 72 °C for 10 min. The PCR product was resolved on 1% agarose gel and sequenced using Sanger sequencing at a commercial facility (Eurofins Genomics India Pvt. Ltd., Bengaluru, India). The obtained sequences were screened using Finch TV v 1.4.0 and searched against NCBI database using homology search (BLASTn). After validation, consensus sequences were deposited at GenBank, an NCBI database, with accession numbers given in Table 2 and Table 3.

### 2.4. Phylogenetic Analysis

Phylogenetic analysis was performed using sequences obtained from the PCR amplification of genetic regions of the *Alternaria* (ITS, LSU, NS and *TEF-α)* and *Colletotrichum* (*TUB*, *CHS*, ITS, *GADPH* and *ACT*) isolates used in the current study and validated representative sequences of different *Alternaria* and *Colletotrichum* species available in the database. Individual sequences were aligned using the MUSCLE algorithm in MEGA XI software [44] and a phylogenetic tree was constructed using the Maximum Likelihood method and the Tamura–Nei model; analysis was performed with 1000 bootstrap replications. Aligned sequences were also concatenated to obtain multi-locus sequences and used for phylogenetic tree construction using the Maximum Likelihood method and the Tamura–Nei model. Multi-locus sequence analysis (MLSA) was performed with 1000 bootstrap replications. A combined dataset of coding and non-coding regions was used in order to maximize the effectiveness of the genetic diversity analysis amongst *Alternaria* and *Colletotrichum* isolates obtained in the current study [19].

### 2.5. Pathogenicity tests

To prove Koch’s postulates, pathogenicity tests for selected isolates of *Alternaria* and *Colletotrichum* were performed in vitro using the mycelial plug inoculation method with some modifications [27]. Briefly, fresh healthy fruits of cv. Bhagawa were collected from bearing orchards (rainy season crop) of the National Research Centre (ICAR) on Pomegranate, Solapur, Maharashtra. Fruits were collected from the orchard where no pesticide sprays were used for the last 25 days and washed with distilled water to remove surface contaminants. Fruits were further disinfected with 70% ethanol and placed on plastic mesh platforms inside sterile glass jar chambers (diameter × height: 30 cm × 22.5 cm). Sterile distilled water was added underneath the platform (12–15 cm) to maintain high humidity (>70 %). Five fruits were inoculated (per isolate) with mycelial plugs (4 mm) at wounded and non-wounded sites from seven-day-old cultures grown on PDA. Healthy fruits were inoculated with PDA alone, which served as the control (Supplementary Figure S1). The inoculated fruits were incubated at room temperature for 7 days and observed regularly for onset of the symptoms.

## 3. Results and Discussion

### 3.1. Symptoms and Disease Incidence

**Figure 1:**
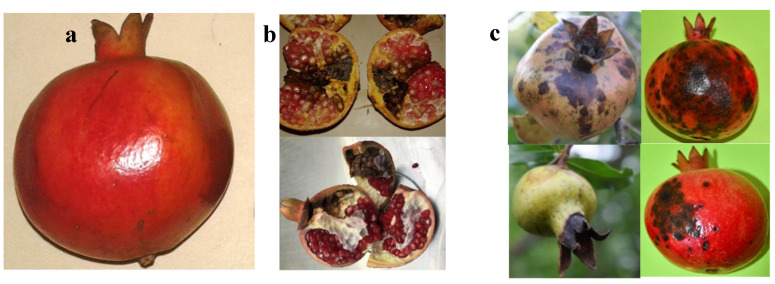
Fruits showing characteristic symptoms of heart rot caused by *Alternaria* (**a**,**b**) and Calyx rot/fruit rot caused by *Colletotrichum* (**c**).

Fungal pathogens belonging to the genus *Alternaria* and *Colletotrichum* cause disease on several horticultural plant species [28,45,46,47,48]. They have been reported to be destructive pathogens infecting pomegranate worldwide [6,25,28,49,50]. *Alternaria* causes heart rot of fruits and leafspot/blight, while *Colletotrichum* causes fruit anthracnose. In the current study, fruits with natural infections of *Alternaria* collected in various regions of India did not exhibit any damage or signs of rotting on the outer surface of the peel; however, they exhibited a peculiar external coloration of the peel (Figure 1a). When such fruits were cut open, the arils inside were brown and rotten (Figure 1b). The infected fruits at advanced stage produced a hollow sound when knocked, while healthy fruits did not. Moreover, infected fruits were lighter than healthy fruits of comparable size and age. These symptoms are characteristic of heart rot of pomegranate [25], and as such it may be difficult to identify heart rot visibly externally. Fruits infected with *Colletotrichum* exhibited characteristic brown-tan hard spots on the surface (Figure 1c). The lesions often displayed gray-orange fungal spore masses on the surface and expanded into the rind and arils, leading to fruit decay [51].

As per yearly data recorded in field surveys at the National Research Centre (ICAR) from 2015 to 2019, the incidence of pomegranate fruit rot ranged from 0 to 8% in the case of *Alternaria* and 0–27% in the case of *Colletotrichum.* However, a remarkable increase was observed in disease incidence in the last two years (2020–2022): up to 25% for *Alternaria* rot and 63% for *Colletotrichum* rot. During these surveys, infected samples were collected from pomegranate orchards in different geographical locations in India, from which around 30 and 45 isolates of *Alternaria* and *Colletotrichum,* respectively, were recovered. Out of these isolates, 12 *Alternaria* and 19 *Colletotrichum* isolates representing different geographical regions were selected based on their morphotypes (Table 2 and Supplementary Table S1). Most of the selected isolates were from Maharashtra, the leading area for pomegranate production and export.

### 3.2. Morphological Characterization of Isolates

**Figure 2:**
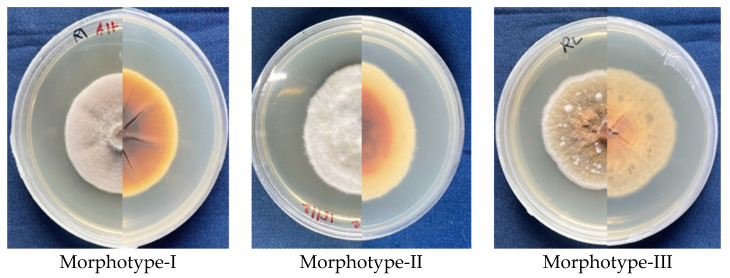
Representative morphotypes of *Alternaria* isolates characterized in the current study. Front and back of seven-day-old colonies grown on PDA at 25 °C.

Colonies produced by *Alternaria* and *Colletotrichum* isolates, when cultured on PDA, displayed intra-genus variability of morphological features. *Alternaria* isolates could be broadly grouped into three morphotypes based on colony morphology (Figure 2). Morphotype I: colonies that appeared greyish white on the obverse; on the reverse, the innermost part was dark brown with brownish white margins. Eight isolates (Alt-1, 3, 5, 6, 7, 8, JD and N) belonged to this morphotype. Morphotype II: colonies that appeared white on the obverse and light-brown with a white margin on the reverse. Isolates Alt-12 and 13 belonged to this morphotype. Morphotype III: colonies that appeared brownish on the obverse and light-brown with a white margin on the reverse. Isolates Alt-2 and MP6 belonged to this morphotype. The color of colonies (grey–white–brown) produced by *Alternaria* isolates in the current study (Supplementary Table S2) are characteristic of this genus [52,53]. The growth rate of different isolates ranged from 4.5 to 7 mm per day, with a maximum exhibited by isolate Alt-8 and a minimum by isolate Alt-13 (Table 4).

**Figure 3:**
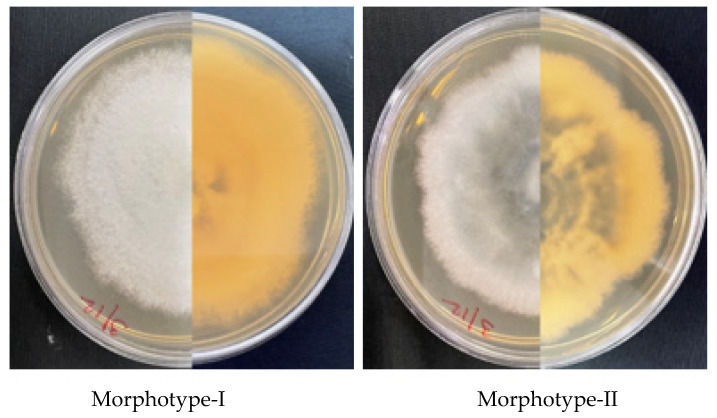
Representative morphotypes of *Colletotrichum* isolates characterized in the current study. Front and back of seven-day-old colonies grown on PDA at 25 °C.

Conversely, *Colletotrichum* isolates could be broadly grouped into two morphotypes (Figure 3). Morphotype I comprised isolates that produced colonies which appeared white and fluffy (Col-1, 4, 11, 18, and 21), mostly with regular margins on the obverse and pale-yellow to yellow on the reverse. The majority of isolates (Col-2, 3, 6, 7, 8, 9, 12, 13, 14, 15, 17, 25, 26 and 27) belonged to morphotype II, which produced colonies that appeared grey in the middle with whitish irregular margins on the obverse and greyish yellow on the reverse. Such morphological features of colonies produced by *Colletotrichum* isolates in the current study (Supplementary Table S3) are characteristic of *Colletotrichum* as reported in previous studies by other authors [27,51]. The growth rate of different isolates ranged from 3.29 to 11.14 mm per day (Table 4).

**Figure 4:**
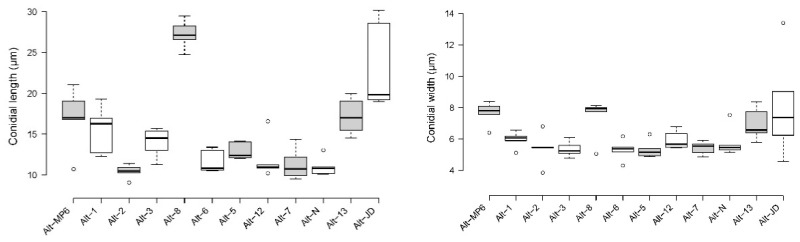
Box plots showing the variation in length and width of conidia produced by *Alternaria* isolates characterized in the current study. Center lines show the medians; box limits indicate the 25th and 75th percentiles, as determined by R software; whiskers extend 1.5 times the interquartile range from the 25th and 75th percentiles; outliers are represented by dots. n = 5 sample points.

Conidia produced by the majority of *Alternaria* isolates were ovoid, with only two isolates (Alt-8 and Alt-13) producing obclavate conidia, and appeared light-to-dark-brown under the microscope. Conidial length ranged from 11 to 27 µm, width from 5 to 8 µm, while the beak length varied from 1.9 to 3.7 µm (Supplementary Table S4). Conidia produced by Alt-2 were the shortest and those by Alt-8 were the longest (Figure 4). However, the beak length was shortest in the Alt-7 isolate and longest in the Alt-JD isolate. The dimensions of conidia, as observed in the current study, corresponded to those reported previously for small spore species of *Alternaria* [54]. The number of horizontal septa ranged from 2 to 5, while the number of vertical septa ranged from 0 to 2 amongst the isolates characterized in this study (Supplementary Table S4).

**Figure 5:**
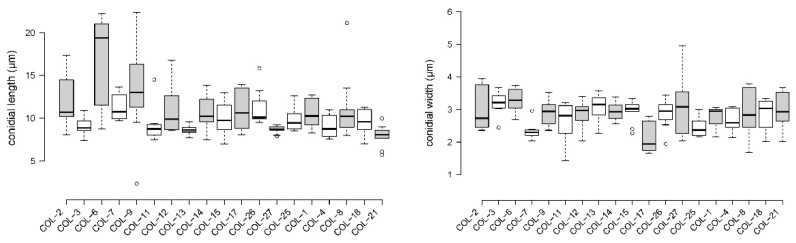
Box plots showing the variation in length and width of conidia produced by *Colletotrichum* isolates characterized in the current study. Center lines show the medians; box limits indicate the 25th and 75th percentiles, as determined by R software; whiskers extend 1.5 times the interquartile range from the 25th and 75th percentiles; outliers are represented by dots. *n* = 5 sample points.

*Colletotrichum* isolates produced cylindrical, hyaline, single-celled conidia. Conidia of isolates Col-1, 2, 3, 9, 11, 12, 13, 14, 15, 17, 18 and 25 were rounded at both ends, while conidia of the rest of the isolates were pointed at one end and rounded at the other. Conidial length ranged from 7.9 to 17 µm and width from 2.1 to 3.2 µm, with a length-to-width ratio ranging between 2.7 and 5.3 (Figure 5, Supplementary Table S5).

**Figure 6:**
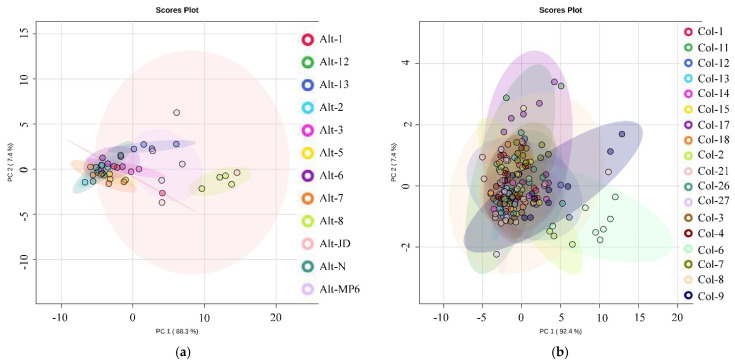
Principal component analysis (PCA) of conidial features of isolates of *Alternaria* (**a**) and *Colletotrichum* (**b**).

Principal component analysis (PCA) was performed using conidial features for isolates of both *Alternaria* and *Colletotrichum*. However, based on these characteristics, no clustering or grouping could be obtained (Figure 6), confirming that morphological features alone are not sufficient to separate and identify the isolates [55]. Delimiting species boundaries and accurate identification of species belonging to the *Alternaria* genus within the *Alternaria* section is difficult because of the overlapping of characteristics and morphological plasticity under different cultural conditions [56]. Therefore, morphological characterization accompanied by molecular information has been utilized for species identification in *Alternaria* species [57]. Similarly, for *Colletotrichum*, the morphological features are not reliable for identifying species and defining species boundaries. Therefore, other features based on DNA/RNA sequences, secondary metabolite production or pathogenicity have been employed either alone or in combination for accurate identification of *Colletotrichum* species [16].

### 3.3. Molecular Characterization

Despite grouping into different morphotypes based on colony morphology, conidial features could not group the isolates. Since species belonging to *Alternaria* or *Colletotrichum* could not be adequately discriminated using morphological features alone, molecular approaches were also used. Individual genetic regions that were PCR amplified and sequenced were searched against the NCBI database. However, based on the BLASTn results, no single species could be deduced, and thus only genera were confirmed based on a homology search. Multi-locus phylogeny was applied for resolving such ambiguity at the species identification level. In particular, ITS, LSU, NS and *TEF-α* regions were targeted for identifying species of *Alternaria*, and ITS*, ACT* and *GAPDH* for *Colletotrichum* species (Table 1). Phylogenetic trees were drawn for individual fragments; however, the trees were not congruent (Supplementary Figures S2–S7). To resolve the incongruence posed by single-gene phylogeny, a combined dataset of coding and non-coding regions was used in order to maximize the effectiveness of the genetic diversity analysis amongst *Alternaria* and *Colletotrichum* isolates obtained in the current study. Since *NS* is not able to provide much information useful for species identification [56], it was excluded from the combined dataset.

### 3.4. Phylogenetic Analyses

**Figure 7:**
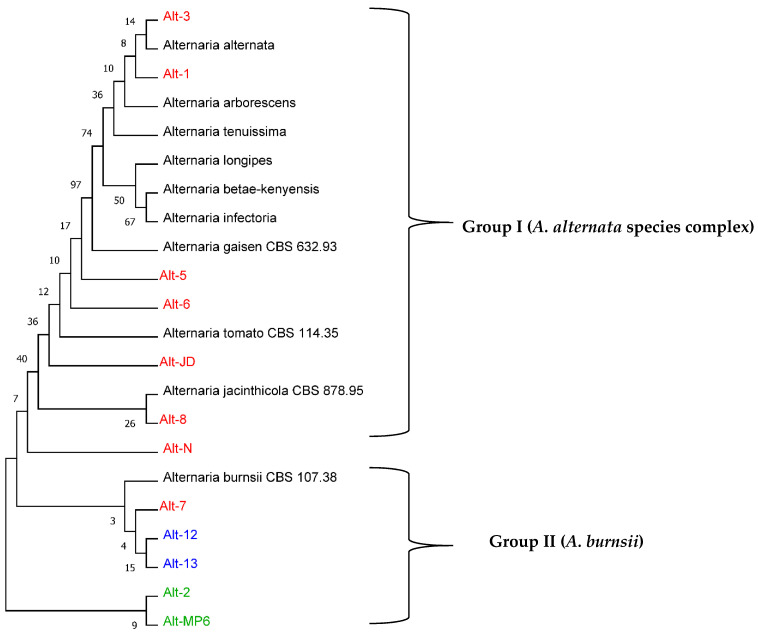
Multi-locus sequence analysis (MLSA) using three genomic regions (ITS, LSU and *TEF-α*) of different isolates of *Alternaria* characterized in the current study. The Maximum Likelihood tree was drawn using MEGA XI with 1000 bootstraps. Isolates in red were of morphotype I, those in blue of morphotype II and those in green of morphotype III.

Based on the result of multi-locus phylogenetic analysis, *Alternaria* isolates clustered into two groups: group I, containing seven isolates (Alt-1, 3, 5, 6, 8, JD and N), showing close relatedness with *Alternaria* species belonging to the *A. alternata* species complex; and group II, containing five isolates (Alt-2, 7, 12, 13 and MP6), showing close relatedness to *A. burnsii* (Figure 7). All isolates of morphotype I, except Alt-7, were in group I, while isolates of morphotype II and III were in group II, indicating a partial overlap between colony morphology and multi-gene phylogeny. The presence of *A. arborescens* close to *A. alternata* in group I could be due to inconsistencies related to the *A. arborescens* species complex (AASC) reported in the literature [22,56,58,59]. For example, some studies have reported AASC to be distinct from the *A. alternata* species complex, while others have identified AASC as a subspecies of *A. alternata* or a different morphotype. However, *A. arborescens* has been retained in *Alternaria* sect. *Alternaria* [56]. Consistently, the isolates indicated under group I in the current study were referred to *Alternaria* sect. *Alternaria.* Moreover, a number of species of *Alternaria*, including *A. solani*, have been reported to infect pomegranate [22,28,60]; however, many of these species were not represented among the isolates characterized in the current study. *Alternaria* species, due to their high adaptivity to different environmental conditions, can infect pomegranate fruits in both pre- and post-harvest stages [61]; they produce toxins which are important for their virulence and may contaminate fruits and the products processed downstream [22]. Moreover, some species, such as *A. gaisen*, are quarantine pathogens imposing export restrictions, and therefore accurate identification of *Alternaria* species also has toxicological and phytosanitary implications.

**Figure 8:**
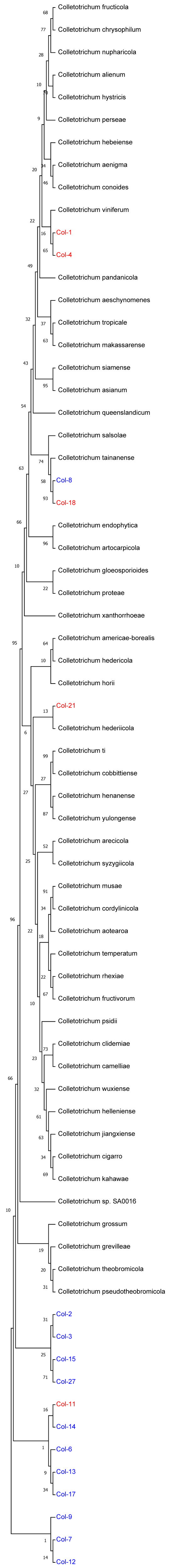
Multi-locus sequence analysis based on five genomic regions (*TUB, CHS,* ITS*, ACT* and *GAPDH*) amplified from different isolates of *Colletotrichum* obtained from pomegranate in India and reference isolates of diverse species belonging to the *Colletotrichum gloeosporioides* species complex. The Maximum Likelihood (ML) tree was drawn using MEGA XI with 1000 bootstraps. Isolates in red belong to morphotype I, while those in blue to morphotype II.

For multi-locus phylogeny of *Colletotrichum* isolates, sequences for species belonging to the *Colletotrichum gloeosporioides* species complex were retrieved [62]. In total, 52 species were included in the analysis, along with 17 isolates characterized in the current study. The combined dataset obtained by concatenation of five loci were used, and in case the sequence was not available, gaps were used in the alignment. As per the ML tree, the isolates belonging to morphotype I resolved well; for example, Col-1 and Col-4 showed close proximity to *C. viniferum*, Col-8 and Col-18 clustered together with *Colletotrichum tainanense*, while Col-21 grouped with *Colletotrichum hederiicola*. On the other hand, isolates belonging to morphotype II formed a separate cluster, showing closeness to the cluster containing *Colletotrichum theobromicola* and *C. pseudotheobromicola.* This cluster also contained Col-11, which belonged to morphotype I (Figure 8). *Colletotrichum* is one of the most economically important and highly damaging genus of plant pathogens and comprises several species complexes [62]. *Colletotrichum gloeosporioides*, long considered as a single species, is now regarded as one of the 14 *Colletotrichum* species complexes and encompasses 22 species [63,64,65,66,67]. In our study, some of the isolates showed close association with *C. theobromicola*, while others grouped with other species in the *C. gloeosporioides* species complex, indicating high genetic variability amongst them. Diversity of *Colletotrichum* species associated with the same host plant has also been reported for many anthracnose diseases of horticultural crops, such as citrus and olive, to name a few [15,68,69,70,71].

Since different *Alternaria* and *Colletotrichum* species have different sensitivity toward the commercially available and routinely applied fungicides [10,11,12,72,73], the complete and precise knowledge of the species involved in the etiology of heart rot and anthracnose of pomegranate fruits is the first step toward devising effective management strategies aimed at preventing these two major fungal diseases of pomegranate in India.

### 3.5. Pathogenicity Tests

Representative isolates were tested for their pathogenicity on detached fruits. *Alternaria* isolates obtained from both fruits and leaves induced rotting on the surface of fruit peel, as well as inside the fruit in the arils, 12 days after inoculation (Supplementary Figure S1). *Alternaria alternata* isolates produced both types of symptoms, while other species of *Alternaria* caused internal rotting only. All *Colletotrichum* isolates induced characteristic symptoms of anthracnose 12–15 days after inoculation (Supplementary Figure S1). Thus, Koch’s postulates were verified for both the pathogens.

## 4. Conclusions

The present study highlighted the variability of *Alternaria* and *Colletotrichum* associated with pomegranate in India and showed that heart rot and anthracnose of pomegranate fruits are caused by diverse *Alternaria* and *Colletotrichum* species, respectively. Most of these species were reported previously in other pomegranate-growing countries. However, many of the taxa identified in this study are first records on pomegranate in India, and *A. burnsii* is reported for the first time as a pathogen of pomegranate worldwide.

## Figures and Tables

**Table 1:** Primers used for DNA amplification in the molecular characterization of *Alternaria* and *Colletotrichum* isolates.

Region Amplified #	Primer Sequences (5′-3′)	Amplicon Size (bp)	Reference
ITSa, c	ITS 1: TCTGTAGGTGAACCTGCGGG ITS-4: TCCTCCGCTTA TTGATATGC	615	[39]
LSUa	LROR: ACCCGCTGAACTTAAGC LR5: TCCTGAGGGAAACTTCG	986	[40]
NSa	NS1: GTAGTCATATGCTTGTCT NS4: CTTCCGTCAATTCCCTTTAAG	1043	[39]
TEF-αa	EF-F: GCYCCYGGHCAYCGTGAYTTYAT EF-R: ACHGTRCCRATACCACCRATCTTT	650	[41]
ACTc	ACT512F: ATGTGCAAGGCCGGTTTCGC ACT783R: TACGAGTCCTTCTGGCCCAT	230	[42]
GAPDHc	GDF1: GCCGTCAACGACCCCTTCATTGA GDR1: GGGTGGAGTCGTACTTGAGCATGT	250	[43]

# ITS: Internal Transcribed Spacer; LSU: Larger subunit; NS: Smaller subunit; TEF-α: Translation Elongation Factor-α; ACT: Actin; GAPDH: Glyceraldehyde-3-phosphate dehydrogenase; a: fragments amplified in *Alternaria* isolates, c: fragments amplified in *Colletotrichum* isolates.

**Table 2:** Geographical origin and accession numbers of gene sequences of *Alternaria* isolates from pomegranate fruits characterized in the current study.

Isolate Code	Geographical Location	Accession Numbers of Gene Sequences			
		ITS	LSU	NS	TEF-α
Alt-5	Kanpur, UP	OL662873	OM073988	OM108305	ON971380
Alt-1	Solapur, MH	OL662874	OM073989	-	ON993381
Alt-6	Aurangabad, MH	OL662875	OM073990	OM108306	ON993382
Alt-7	Solapur, MH	OL662876	OM073991	OM108307	ON993383
Alt-12	Nanded, MH	OL662877	OM073992	OM108308	ON993384
Alt-13	Solapur, MH	OL662878	OM073993	OM108309	ON993385
Alt-JD	Solapur, MH	OL662879	OM073994	OM108310	ON993386
Alt-N	Beed, MH	OL662880	OM073995	OM108311	ON993387
Alt-2	Solapur, MH	OL662881	OM073996	OM108312	ON993388
Alt-3	Parbhani, MH	OL662882	OM073997	OM108313	ON993389
Alt-8	Chhindwara, MP	OL662883	OM073998	OM108314	ON993390
Alt-MP6	Solapur, MH	OL662884	OM073999	OM108315	ON993391

**Table 3:** Geographical origin and accession numbers of gene sequences of *Colletotrichum* isolates from pomegranate fruits characterized in the current study.

Isolate Code	Geographical Location	Accession Numbers of Gene Sequences		
		ITS	ACT	GAPDH
Col-1	Chitradurga, KA	OM638721	ON971379	ON971360
Col-2	Solapur, MH	OM638722	ON993365	ON971361
Col-3	Jalna, MH	OM638723	ON993366	ON971374
Col-4	Jalna, MH	OM638724	ON993367	ON971362
Col-6	Solapur, MH	OM638725	ON993368	ON971363
Col-7	Beed, MH	OM638726	ON993369	ON971364
Col-8	Satara, MH	OM638727	ON993370	ON971375
Col-9	Nandurbar, MH	OM638728	ON993371	ON971365
Col-11	Selam, TN	OM638729	ON993372	ON971366
Col-12	Solapur, MH	OM638730	ON993373	ON971376
Col-13	Jalna, MH	OM638731	ON993374	ON971367
Col-14	Solapur, MH	OM638732	ON993375	ON971368
Col-15	Solapur, MH	OM638733	ON993376	ON971377
Col-17	Solapur, MH	OM638734	ON993377	ON971369
Col-18	Solapur, MH	OM638735	ON993378	ON971370
Col-21	Bagalkot, KA	ON908458	ON993379	ON971378
Col-25	Solapur, MH	ON908459	-	ON971371
Col-26	Solapur, MH	OM638736	-	ON971372
Col-27	Solapur, MH	ON908460	ON993380	ON971373

**Table 4:** Mean growth rate of isolates of diverse species of *Alternaria* (*n* = 12) and *Colletotrichum* (*n* = 19) from pomegranate characterized in this study determined after seven days incubation on PDA at 25 °C.

Isolate Name	Species	Growth Rate (mm/day) ^a^	Isolate Name	Species	Growth Rate (mm/day) ^a^
*Alternaria* Isolates			*Colletotrichum* Isolates		
Alt-1	*A. alternata*	6.64 ± 0.5	Col-1	*C. viniferum*	10.86 ± 0.14
Alt-2	*A. burnsii*	6.79 ± 0.07	Col-2	*C. gloeosporioides*	10.71 ± 0.29
Alt-3	*A. alternata*	5.64 ± 0.07	Col-3	*C. gloeosporioides*	5.57 ± 0.14
Alt-5	*A. alternata*	5.96 ± 0.39	Col-4	*C. viniferum*	5.86 ± 0.0
Alt-6	*A. alternata*	6.93 ± 0.5	Col-6	*C. gloeosporioides*	5.86 ± 0.0
Alt-7	*A. burnsii*	6.71 ± 1.14	Col-7	*C. gloeosporioides*	10.57 ± 0.0
Alt-8	*A. alternata*	7.07 ± 0.07	Col-8	*C. tainanense*	11.14 ± 0.0
Alt-12	*A. burnsii*	6.11 ± 0.68	Col-9	*C. gloeosporioides*	5.29 ± 0.0
Alt-13	*A. burnsii*	4.50 ± 0.5	Col-11	*C. gloeosporioides*	3.86 ± 0.0
Alt-JD	*A. alternata*	4.86 ± 0.86	Col-12	*C. gloeosporioides*	10.00 ± 0.0
Alt-MP6	*A. burnsii*	5.64 ± 0.21	Col-13	*C. gloeosporioides*	10.57 ± 0.0
Alt-N	*A. alternata*	6 ± 1.43	Col-14	*C. gloeosporioides*	9.57 ± 0.43
			Col-15	*C. gloeosporioides*	9.14 ± 0.0
			Col-17	*C. gloeosporioides*	3.29 ± 0.0
			Col-18	*C. tainanense*	5.29 ± 0.14
			Col-21	*C. hederiicola*	5.43 ± 0.0
			Col-25	*C. gloeosporioides*	3.29 ± 0.0
			Col-26	*C. gloeosporioides*	5.14 ± 0.0
			Col-27	*C. gloeosporioides*	9.86 ± 0.14

^a^ Means of three replicates ± SD.

## Data Availability

The authors confirm that the data supporting the findings of this study are available within the article [and/or] its Supplementary Materials.

## Supplementary Materials

The following supporting information can be downloaded at: https://www.mdpi.com/article/10.3390/jof8101040/s1, Figure S1: Flowchart depicting steps performed during pathogenicity tests. Figure S2: Phylogenetic analysis using elongation factor (*TEF-α*) gene amplified and sequenced from *Alternaria* isolates. Maximum Likelihood tree drawn using MEGA XI with 1000 bootstraps. Figure S3: Phylogenetic analysis using ITS region amplified and sequenced from *Alternaria* isolates. Maximum Likelihood tree drawn using MEGA XI with 1000 bootstraps. Figure S4: Phylogenetic analysis using nuclear ribosomal large subunit (LSU) region amplified and sequenced from *Alternaria* isolates. Maximum Likelihood tree drawn using MEGA XI with 1000 bootstraps. Figure S5: Phylogenetic analysis using actin gene amplified and sequenced from *Colletotrichum* isolates. Maximum Likelihood tree drawn using MEGA XI with 1000 bootstraps. Figure S6: Phylogenetic analysis using *GAPDH* (glyceraldehyde phosphate dehydrogenase) gene amplified and sequenced from *Colletotrichum* isolates. Maximum Likelihood tree drawn using MEGA XI with 1000 bootstraps. Figure S7: Phylogenetic analysis using ITS region amplified and sequenced from *Colletotrichum* isolates. Maximum Likelihood tree drawn using MEGA XI with 1000 bootstraps. Table S1: Origin of isolates of *Alternaria* (n = 12) and *Colletotrichum* (n = 19) from pomegranate characterized in this study. Table S2: Morphotypes of *Alternaria* isolates (n = 12) characterized in the current study. Front and back of seven-day-old colonies grown on PDA at 25 °C. Table S3: Morphotypes of *Colletotrichum* isolates (n = 19) characterized in the current study. Front and back of seven-day-old colonies grown on PDA at 25 °C. Table S4: Microscopic features of conidia produced by different isolates (n = 12) of *Alternaria* characterized in this study. Table S5: Microscopic features of conidia produced by different isolates (n = 19) of *Colletotrichum* characterized in this study.
