# Supplementary material for: Characterization of Alternaria and Colletotrichum Species Associated with Pomegranate (Punica granatum L.) in Maharashtra State of India
Source: J Fungi (Basel). 2022 Sep 30;8(10):1040. doi: 10.3390/jof8101040 (PMC9604645; doi:10.3390/jof8101040)
Supplement: Supplementary file 1 [file jof-08-01040-s001.zip › jof-1859640-supplementary/supplementary figures.pdf]

Supplementary figures:

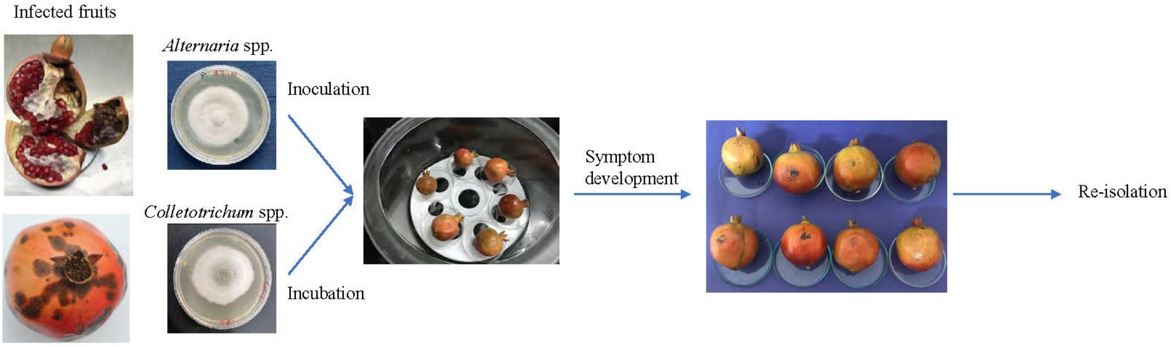

Figure S1. Flowchart depicting steps performed during pathogenicity tests

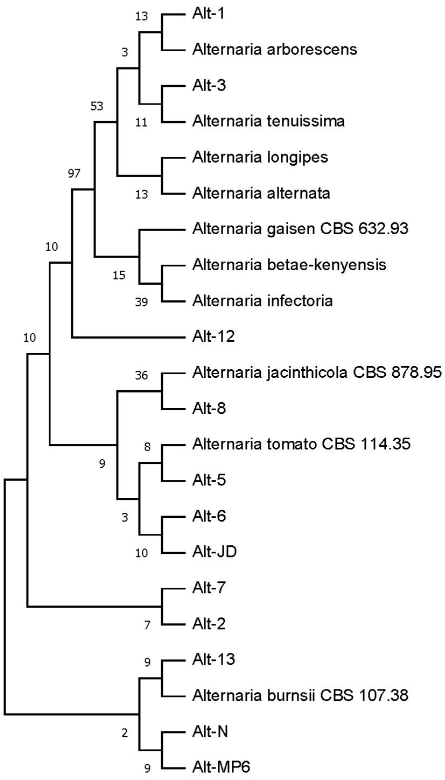

Figure S2. Phylogenetic analysis using elongation factor (*TEF-α*) gene amplified and sequenced from *Alternaria* isolates. Maximum Likelihood tree drawn using MEGA XI with 1000 bootstrap

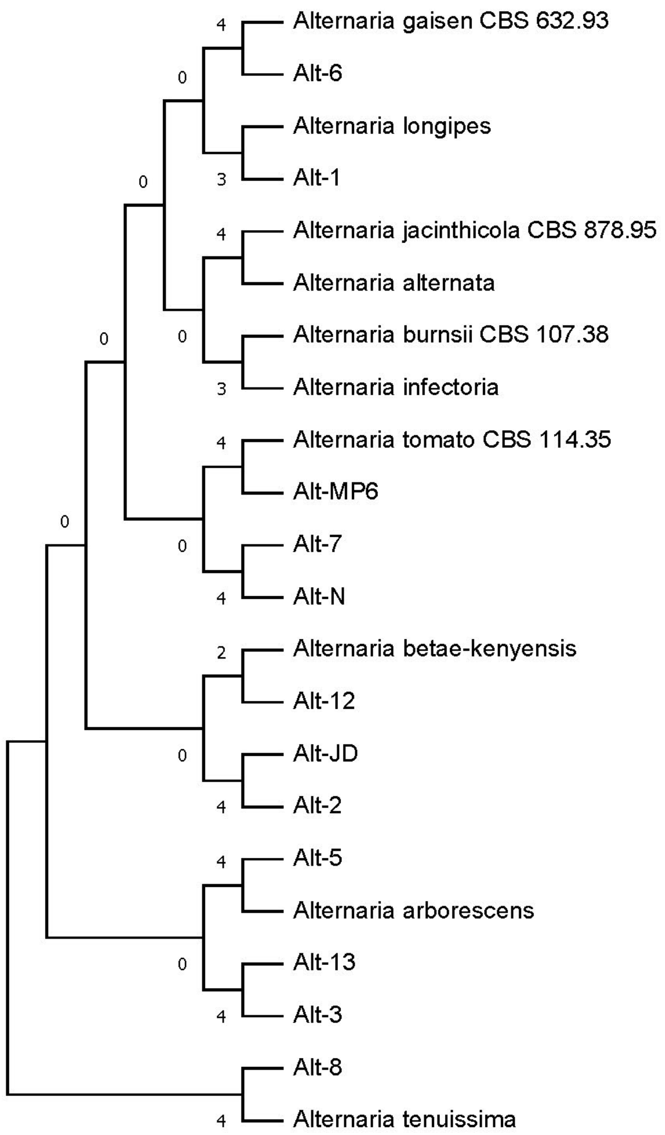

Figure S3. Phylogenetic analysis using *ITS* region amplified and sequenced from *Alternaria* isolates. Maximum Likelihood tree drawn using MEGA XI with 1000 bootstrap

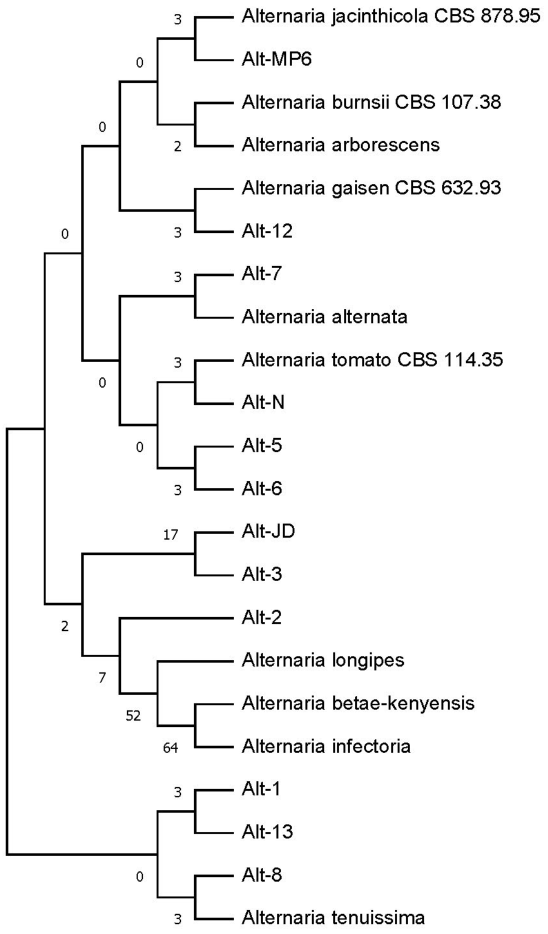

Figure S4. Phylogenetic analysis using nuclear ribosomal large subunit (LSU) region amplified and sequenced from *Alternaria* isolates. Maximum Likelihood tree drawn using MEGA XI with 1000 bootstrap
