# Supplementary material for: Characterization of Alternaria and Colletotrichum Species Associated with Pomegranate (Punica granatum L.) in Maharashtra State of India
Source: J Fungi (Basel). 2022 Sep 30;8(10):1040. doi: 10.3390/jof8101040 (PMC9604645; doi:10.3390/jof8101040)
Supplement: Supplementary file 1 [file jof-08-01040-s001.zip › jof-1859640-supplementary/Supplementary_Tables round 2.pdf]

Supplementary Table S1. Origin of isolates of *Alternaria* (n=12) and *Colletotrichum* (n=19) from pomegranate characterized in this study

Isolate Name	Taluk & District	Co-ordinates	State	Year of collection	Source of isolation
***Alternaria* isolates**					
Alt-1	Kanpur	26°19'97.053"N 79°84'41.634"E	Uttar Pradesh	2016	Leaf
Alt-2	Malshiras, Solapur	17°70'23.773"N 75°08'62.023"E	Maharashtra	2016	Fruit
Alt-3	Jalna, Aurangabad	19°50'07.875"N 75°58'64.147"E	Maharashtra	2016	Fruit
Alt-5	Mangalwedha, Solapur	17°30'41.057"N 75°27'06.779"E	Maharashtra	2017	Fruit
Alt-6	Nanded	19°08'17"N 77°19'15"E	Maharashtra	2019	Fruit
Alt-7	Madha, Solapur	17°95'24.105"N 75°59'79.319"E	Maharashtra	2019	Fruit
Alt-8	Sangola, Solapur	17°20'68.107"N 75°00'60.615"E	Maharashtra	2019	Fruit
Alt-12	Beed	18°35'32.6"N 75°27'04.0"E	Maharashtra	2020	Fruit
Alt-13	Sangola, Solapur	17°26'05.458"N 75°11'58.566"E	Maharashtra	2020	fruit
Alt-JD	Parbhani	19°09'14.0"N 76°27'46.4"E	Maharashtra	2021	Fruit
Alt-MP6	Chhindwara	22°89'59"N 77°75'78"E	Madhya Pradesh	2021	Fruit
Alt-N	NRCP, Solapur	17°70'62.443"N 75°83'38.268"E	Maharashtra	2021	Fruit
***Colletotrichum* isolates**					
Col-1	Chitradurga	13°47'54.6936"N 76°17'06.2052"E	Karnataka	2015	fruit
Col-2	Solapur	17°53'55.014"N 74°45'15.0804"E	Maharashtra	2015	fruit
Col-3	Jalna	19°26'23.3448"N 75°55'24.3336"E	Maharashtra	2015	fruit
Col-4	Jalna	19°50'04.7976"N 75°52'53.8824"E	Maharashtra	2015	fruit
Col-6	Solapur	17°07'28.0164"N 74°11'14.622"E	Maharashtra	-	fruit
Col-7	Beed	19°11'11.6628"N 76°13'29.0532"E	Maharashtra	2015	fruit
Col-8	Satara	18°02'30.1416"N 74°11'10.2552"E	Maharashtra	2015	fruit
Col-9	Nandubar	N 21°21'51.5988"N 74°17'52.3968"E	Maharashtra	2015	fruit
Col-11	Selam	N 11°07'37.6392"N 78°39'24.8184"E	Tamil Nadu	2015	fruit
Col-12	Solapur	N 17°51'47.718"N 74°54'19.7244"E	Maharashtra	2015	fruit
Col-13	Jalna	N 19°50'04.7976"N 75°52'53.8824"E	Maharashtra	2016	fruit
Col-14	Solapur	N 17°41'20.9616"N 75°48'20.9484"E	Maharashtra	2017	fruit
Col-15	Solapur	N 17°33'55.3644"N 75°34'19.4304"E	Maharashtra	2017	fruit

Col-17	Solapur	N 17°39'35.7084"N 75°54'23.0076"E	Maharashtra	2017	fruit
Col-18	Solapur	N 17°51'47.718"N 74°54'19.7244"E	Maharashtra	2017	fruit
Col-21	Karnataka	N 15°19'02.1972"N 75°42'49.9968"E	Karnataka	2018	fruit
Col-25	Solapur	N 18°18'13.7952"N 75°09'58.3632"E	Maharashtra	2019	fruit
Col-26	Hiraj, Solapur	N 17°41'20.9616"N 75°48'20.9484"E	Maharashtra	2021	fruit
Col-27	Solapur	N 17°39'35.7084"N 75°54'23.0076"E	Maharashtra	2021	fruit

Supplementary Table S2. Morphotypes of *Alternaria* isolates (n=12) characterized in the current study. Front and back of 7-days- old colonies grown on PDA at 25°C.

Isolate Name	Colony on PDA (obverse)	Colony on PDA (reverse)	Colony appearance on PDA at 25±1	Micrograph of conidia (40X)	Conidia shape
			Morphotype I		
Alt-1	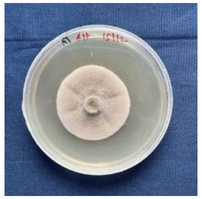	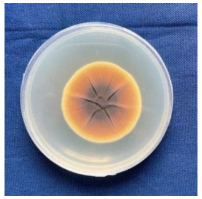	Greyish white on obverse and on reverse, the inner part was dark brown and the margin was brownish white	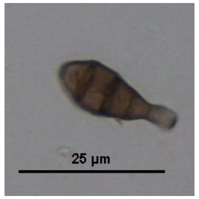	Ovoid
Alt-3	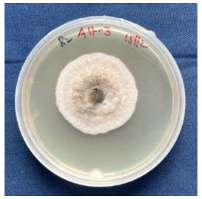	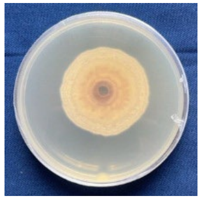	Greyish white with white margin on obverse and on reverse, the innermost part was dark brown and the outer part was brownish	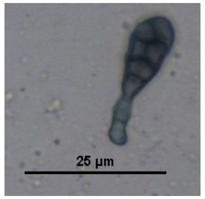	Ovoid

Alt-5	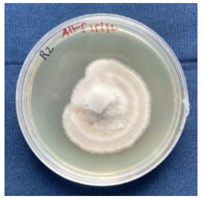	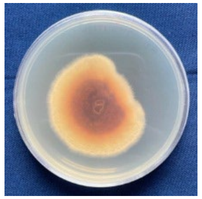	Greyish white on obverse and on reverse, the inner part was dark brown and the outer part was brownish with white margin	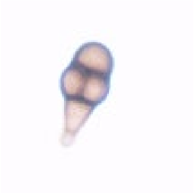	Ovoid
Alt-6	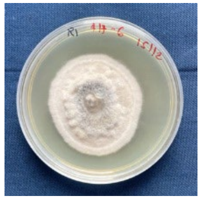	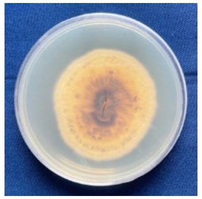	Light grey on obverse and on reverse, the inner part was dark brown and the outer part was light brown with white margin	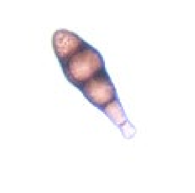	Ovoid
Alt-7	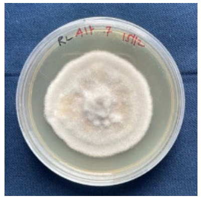	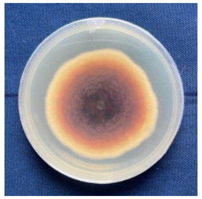	Greyish white on obverse and on reverse, dark brown with brownish white margin	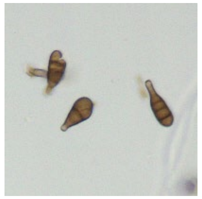	Ovoid
Alt-8	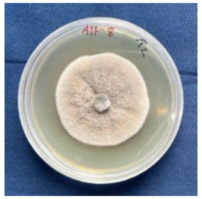	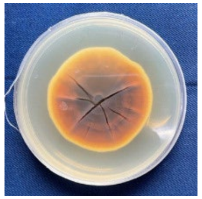	Grey with white margin on obverse and on reverse dark brown with white margin	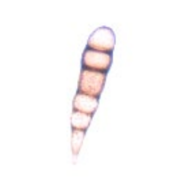	Obclavate

Alt-JD	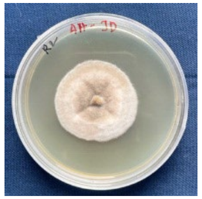	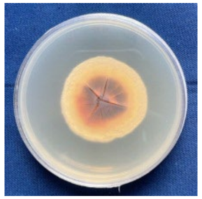	Greyish white on obverse and on reverse, the inner part was dark brown and the margin was brownish white	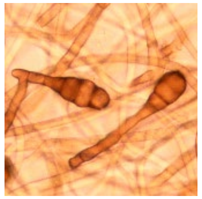	Ovoid
Alt-N	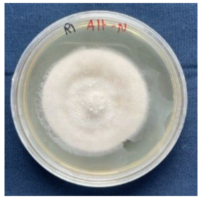	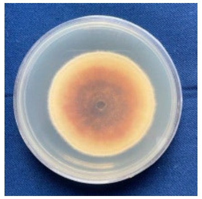	Greyish white on obverse and in reverse, the inner part was dark brown and the margin was brownish white	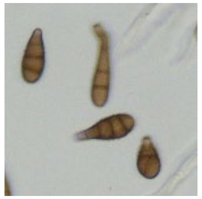	Ovoid
			Morphotype II		
Alt-12	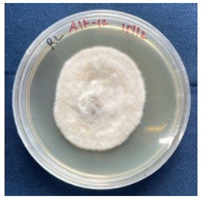	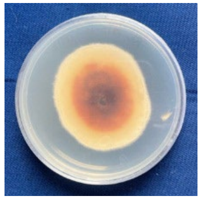	Dull white on obverse and on reverse brown with brownish white margin	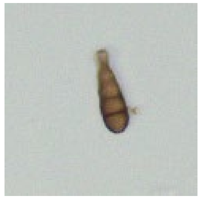	Ovoid
Alt-13	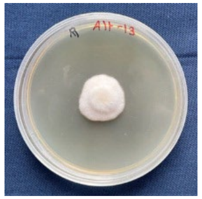	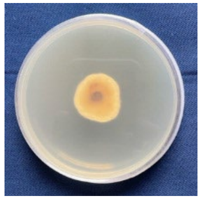	White on obverse and on reverse light brownish with white margin	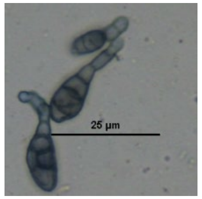	Obclavate
			Morphotype III		

Alt-2	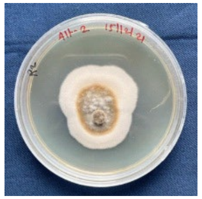	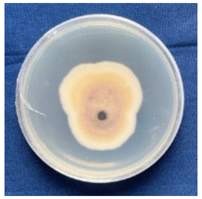	Brownish on obverse and on reverse light brown colour with white margin	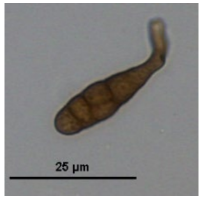	Ovoid
Alt-MP6	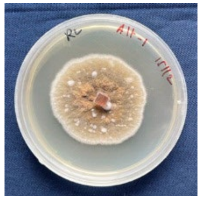	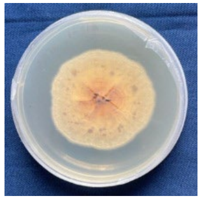	Light brownish on obverse with white margin and in reverse the inner part was brown and outer part was light brown	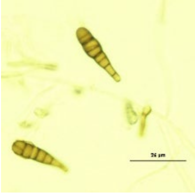	Ovoid

Supplementary Table S3. Morphotypes of *Colletotrichum* isolates (n=19) characterized in the current study. Front and back of 7-days- old colonies grown on PDA at 25°C.

Isolate Name	Colony on PDA (obverse)	Colony on PDA (reverse)	Colony appearance on PDA at 25±1	Micrograph of Conidia (40X)	Conidia shape
			Morphotype I		
Col-1	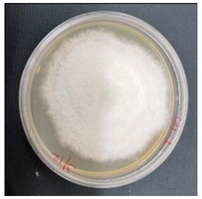	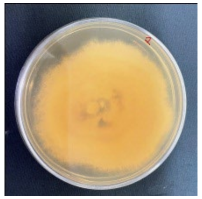	Fluffy white colonies with regular margin, reverse yellow	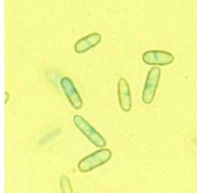	Cylindric, rounded at both ends

Col-4	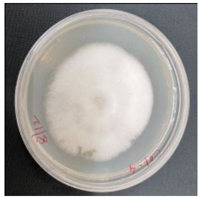	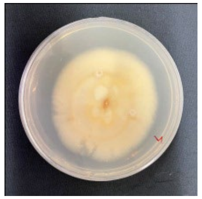	Fluffy white colonies with regular margin, reverse pale yellow	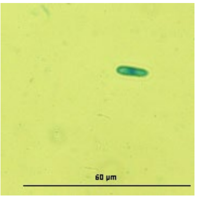	Cylindric, pointed at one end
Col-11	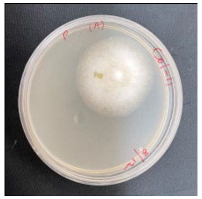	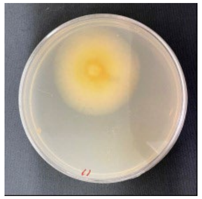	White to pale orange with regular margin, reverse pale ornage	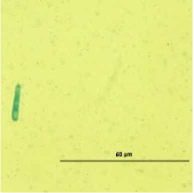	Cylindric, rounded at both ends
Col-18	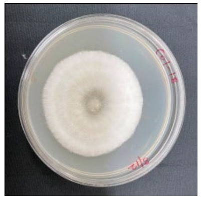	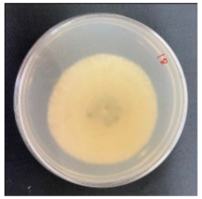	White colonies with regular margin, reverse pale yellow	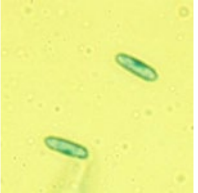	Cylindric, rounded at both ends
Col-21	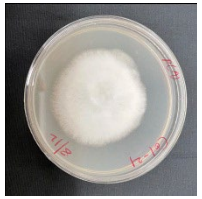	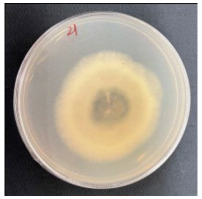	White colonies with regular margin, reverse pale orange	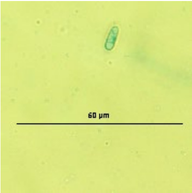	Cylindric, pointed at one end

Morphotype II

Col-2	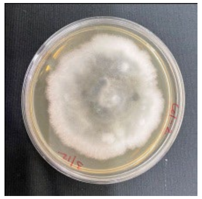	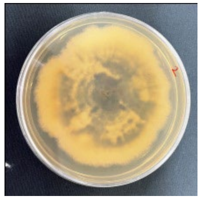	Grey in the middle with whitish irregular margin, reverse greyish yellow	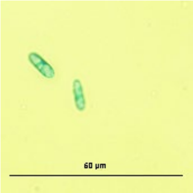	Cylindric, rounded at both ends
Col-3	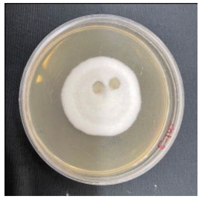	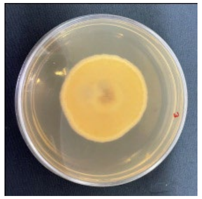	Grey in the middle with whitish regular margin, reverse greyish yellow	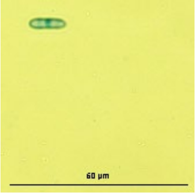	Cylindric, rounded at both ends
Col-6	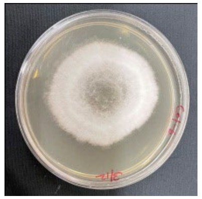	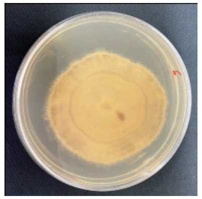	Grey in the middle with whitish regular margin, reverse greyish yellow	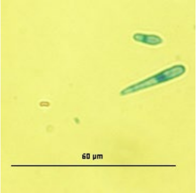	Cylindric, pointed at one end
Col-7	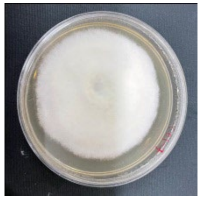	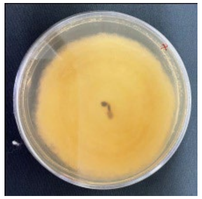	Grey in the middle with whitish regular margin, reverse yellow	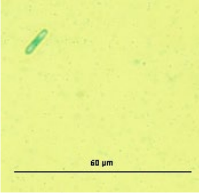	Cylindric, pointed at one end

Col-8	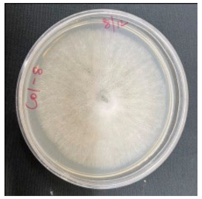	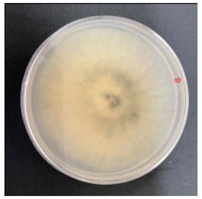	Grey white with regular margin, reverse yellowish brown	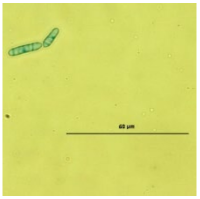	Cylindric pointed at one end
Col-9	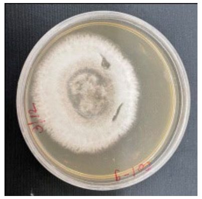	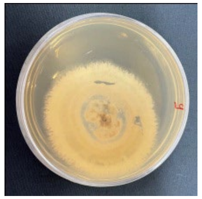	Grey in the middle with whitish regular margin, reverse greyish yellow	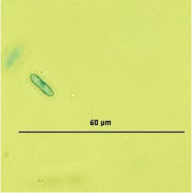	Cylindric, rounded at both ends
Col-12	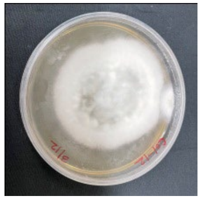	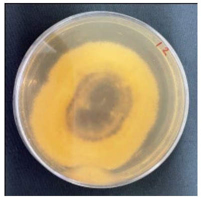	Grey in the middle with whitish regular margin, reverse greyish yellow	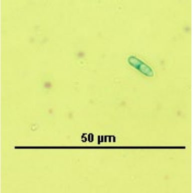	Cylindric, rounded at both ends
Col-13	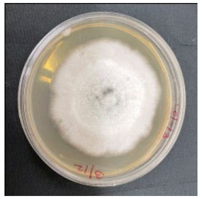	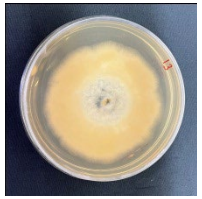	Grey in the middle with whitish irregular margin, reverse greyish yellow	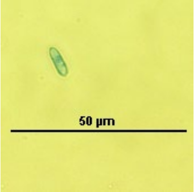	Cylindric, rounded at both ends

Col-14	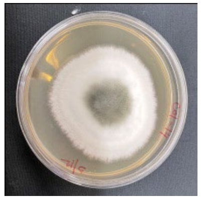	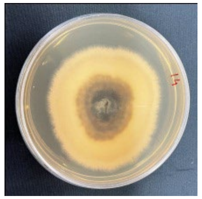	Grey in the middle with whitish regular margin, reverse greyish yellow	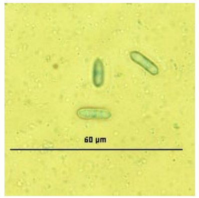	Cylindric, rounded at both ends
Col-15	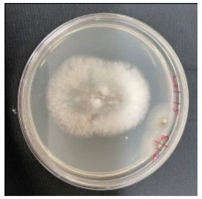	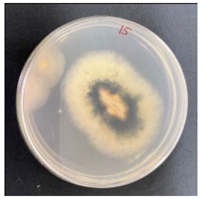	Pale to dark grey with regular margin, reverse yellowish brown	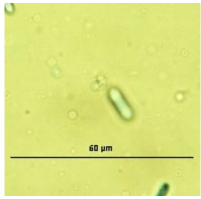	Cylindric, rounded at both ends
Col-17	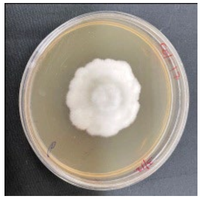	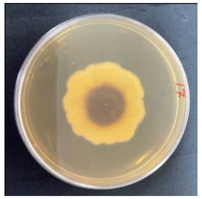	Grey in the middle with whitish irregular margin, reverse greyish yellow	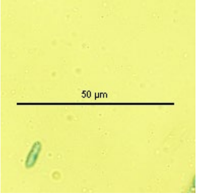	Cylindric, rounded at both ends
Col-25	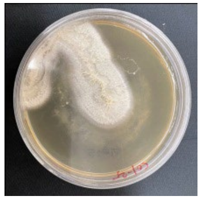	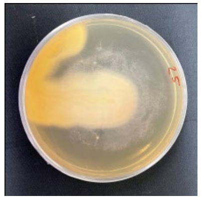	Grey in the middle with whitish irregular margin, reverse greyish yellow	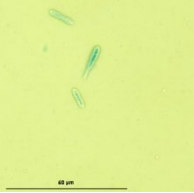	Cylindric, rounded at both ends

Col-26	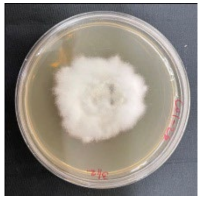	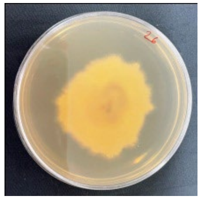	Grey in the middle with whitish irregular margin, reverse greyish yellow	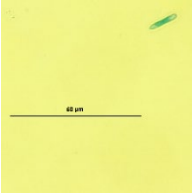	Cylindric, pointed at one end
Col-27	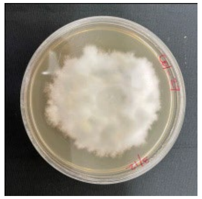	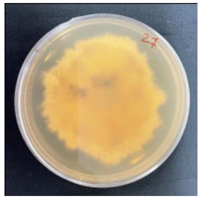	Grey in the middle with whitish irregular margin, reverse greyish yellow	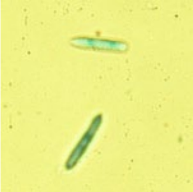	Cylindric, pointed at one end

Supplementary Table S4. Microscopic features of conidia produced by different isolates (n=12) of *Alternaria* characterized in this study.

Isolate Name	**Length***	**Width***	**Beak length***	**Length/Width Ratio**	**Vertical septa**	**Horizontal septa**	**Vertical septa / Horizontal septa Ratio**
Alt-1	15.51±2.65	5.96±0.47	5.7±0.77	2.64±0.64	3.8±0.74	0.6±0.48	2.2±1.83
Alt-12	11.98±2.32	5.94±0.53	4.05±1.25	2.04±0.47	3.2±0.4	0.2±0.4	0.6±1.2
Alt-13	17.21±2.04	6.69±0.93	8.61±1.35	2.47±0.11	2.8±0.74	0.4±0.48	1.4±1.74
Alt-2	10.44±0.78	5.4±0.93	4.3±0.95	1.97±0.24	2.8±0.4	0.2±0.4	0.4±0.8
Alt-3	13.99±1.64	5.3±0.45	5.53±1.15	2.61±0.30	3.2±0.4	0	0
Alt-5	12.96±0.95	5.3±0.51	5.22±1.21	2.43±0.12	2.6±0.8	0	0
Alt-6	11.68±1.27	5.03±0.59	5.94±0.89	2.23±0.33	2.2±0.4	0.2±0.4	0.4±0.8
Alt-7	11.36±1.76	5.42±0.38	3.73±0.59	2.11±0.41	2.2±0.74	0	0
Alt-8	27.24±1.58	7.37±1.16	7.97±0.43	3.77±0.56	5±0.89	0.2±0.4	0.8±1.6
Alt-JD	23.36±4.94	8.12±3.00	8.82±2.62	3.27±1.22	4.2±1.16	0	0

Alt-MP6	16.92±3.46	7.65±0.68	8.09±1.66	2.23±0.52	3.8±1.16	0.8±0.74	1.9±1.85
Alt-N	11.01±1.07	5.8±0.87	4.9±1.30	1.91±0.11	2.2±0.4	0.2±0.4	0.4±0.8

[a]Mean of 5 replicates ± SD. *Length and width measurements are in µm

Supplementary Table S5. Microscopic features of conidia produced by different isolates (n=19) of *Colletotrichum* characterized in this study.

Isolate Name	**Average conidial length* ± SD**	**Average conidial width* ± SD**	**Length/Width Ratio ±SD**
1. Col-1	10.56±1.65	2.8±0.30	3.78±0.53
2. Col-2	11.78±2.70	3.02±0.62	3.89±0.45
3. Col-3	9.06±0.95	3.20±0.33	2.84±0.33
4. Col-4	8.9±1.13	2.634±0.32	3.41±0.69
5. Col-6	17±4.88	3.271±0.34	5.15±1.30
6. Col-7	11.18±1.30	.33±0.23	4.83±0.64
7. Col-8	11.20±3.60	2.88±0.66	4.15±1.67
8. Col-9	13.51±5.43	2.94±0.37	4.80±2.42
9. Col-11	9.15±1.88	2.585±0.58	3.85±1.71
10. Col-12	11.06±2.78	2.88±0.35	3.86±0.91
11. Col-13	8.6±0.48	3.07±0.38	2.86±0.54
12. Col-14	9.78±3.51	2.9±0.23	3.65±0.69
13. Col-15	9.54±1.74	2.94±0.32	3.42±0.70
14. Col-17	11.02±2.06	2.1±0.46	5.31±1.36
15. Col-18	9.54±1.37	2.869±0.44	3.40±0.63
16. Col-21	7.92±1.19	2.98±0.51	2.77±0.52
17. Col-25	10.05±1.45	2.4±0.29	4.06±4.94
18. Col-26	11.19±1.91	2.8±0.40	4.00±1.15
19. Col-27	8.68±0.41	3.1±0.81	2.79±0.51

[a]Mean of 5 replicates ± SD. *Length and width measurements are in µm
