# Supplementary figures and images for: Characterization of Alternaria and Colletotrichum Species Associated with Pomegranate (Punica granatum L.) in Maharashtra State of India
Source: J Fungi (Basel). 2022 Sep 30;8(10):1040. doi: 10.3390/jof8101040 (PMC9604645; doi:10.3390/jof8101040)

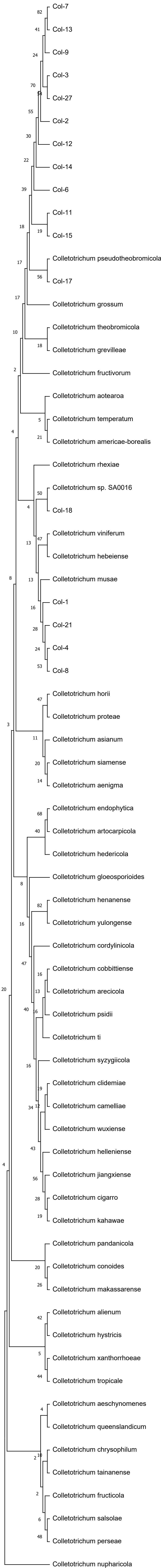

Supplement: Supplementary file 1 [file jof-08-01040-s001.zip › jof-1859640-supplementary/Figure S5 Act.pdf]

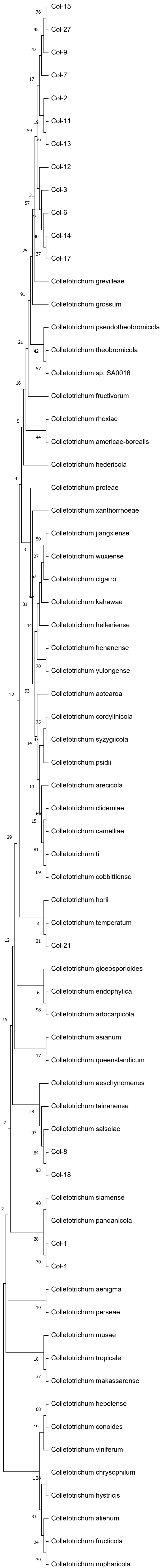

Supplement: Supplementary file 1 [file jof-08-01040-s001.zip › jof-1859640-supplementary/Figure S6 GAPDH.pdf]

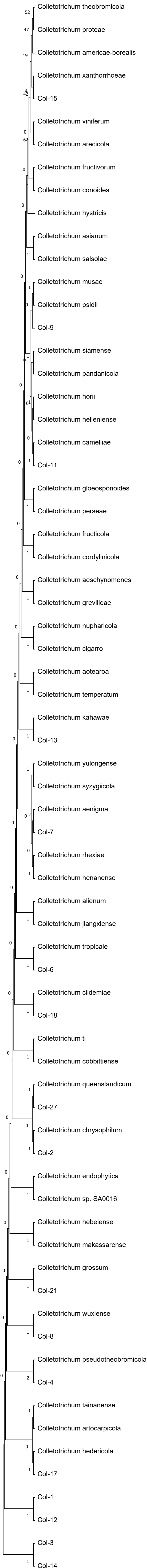

Supplement: Supplementary file 1 [file jof-08-01040-s001.zip › jof-1859640-supplementary/Figure S7 ITS.pdf]
